# Energy Achievement Rate Is an Independent Factor Associated with Intensive Care Unit Mortality in High-Nutritional-Risk Patients with Acute Respiratory Distress Syndrome Requiring Prolonged Prone Positioning Therapy

**DOI:** 10.3390/nu13093176

**Published:** 2021-09-12

**Authors:** Pin-Kuei Fu, Chen-Yu Wang, Wei-Ning Wang, Chiann-Yi Hsu, Shih-Pin Lin, Chen-Tsung Kuo

**Affiliations:** 1 Department of Critical Care Medicine, Taichung Veterans General Hospital, Taichung 407219, Taiwan; chestmen@gmail.com; 2 Ph.D. Program in Translational Medicine, National Chung Hsing University, Taichung 402010, Taiwan; 3 College of Human Science and Social Innovation, Hungkuang University, Taichung 433304, Taiwan; 4 Department of Computer Science, Tunghai University, Taichung 407224, Taiwan; 5 Department of Nursing, Hungkuang University, Taichung 43302, Taiwan; 6 Department of Food and Nutrition, Taichung Veterans General Hospital, Taichung 40705, Taiwan; sherry@vghtc.gov.tw; 7 Biostatistics Task Force of Taichung Veterans General Hospital, Taichung 407219, Taiwan; chiann@vghtc.gov.tw; 8 Department of Information Engineering and Computer Science, Feng Chia University, Taichung 407802, Taiwan; yalebin.lin@gmail.com; 9 Computer & Communications Center, Taipei Veterans General Hospital, Taipei 11217, Taiwan; jmskuo@gmail.com

**Keywords:** acute respiratory distress syndrome, energy achievement rate, high nutritional risk, mortality, modified nutrition risk in the critically ill, prolonged prone positioning

## Abstract

Early enteral nutrition (EN) and a nutrition target >60% are recommended for patients in the intensive care unit (ICU), even for those with acute respiratory distress syndrome (ARDS). Prolonged prone positioning (PP) therapy (>48 h) is the rescue therapy of ARDS, but it may worsen the feeding status because it requires the heavy sedation and total paralysis of patients. Our previous studies demonstrated that energy achievement rate (EAR) >65% was a good prognostic factor in ICU. However, its impact on the mortality of patients with ARDS requiring prolonged PP therapy remains unclear. We retrospectively analyzed 79 patients with high nutritional risk (modified nutrition risk in the critically ill; mNUTRIC score ≥5); and identified factors associated with ICU mortality by using a Cox regression model. Through univariate analysis, mNUTRIC score, comorbid with malignancy, actual energy intake, and EAR (%) were associated with ICU mortality. By multivariate analysis, EAR (%) was a strong predictive factor of ICU mortality (HR: 0.19, 95% CI: 0.07–0.56). EAR >65% was associated with lower 14-day, 28-day, and ICU mortality after adjustment for confounding factors. We suggest early EN and increase EAR >65% may benefit patients with ARDS who required prolonged PP therapy.

## 1. Introduction

Early enteral nutrition (EN) initiated within 48 h is recommended for all critically ill patients treated with invasive mechanical ventilation in the intensive care unit (ICU) [1,2,3,4]. According to current guidelines, a systemic survey of nutritional risk within 24 h of admission is recommended, accompanied by early EN [2,3,4] to reduce the risk of infectious complications and organ failure in critically ill patients [4,5]. The modified nutrition risk in the critically ill (mNUTRIC) score is a powerful screening tool that uses a cutoff value of ≥5 to identify patients with high nutritional risk [6] and even critically ill patients with COVID-19 infection [7]. After the identification of higher-risk groups, the second step is the achievement of feeding goals [1]. The ideal energy achievement rate (EAR) for the first week in the ICU is 60–70% of the nutritional target according to the 2016 American Society for Parenteral and Enteral Nutrition (ASPEN) and Society of Critical Care Medicine (SCCM) guidelines and the 2019 European Society for Clinical Nutrition and Metabolism (ESPN) guidelines [1,3,4]. Our previous studies have also demonstrated that an EAR of >65% was associated with lower mortality risk in medical ICUs [8,9,10,11]. However, the impact of EAR on the mortality of patients with high nutritional risk and acute respiratory distress syndrome (ARDS) requiring prolonged prone positioning (PP) therapy remains unclear.

The most severe condition for patients in medical ICUs is ARDS secondary to pneumonia or sepsis because the mortality rate can vary from 34.9% for mild ARDS to 46.1% for severe ARDS [12,13]. Prolonged PP therapy for at least 16 h per day is the standard of care for moderate to severe ARDS because a landmark study revealed that it reduced mortality [14,15]. PP therapy is an effective strategy to improve oxygenation and secretion clearance in cases of severe COVID-19-associated ARDS (CARDS) [16,17]. However, PP therapy may affect the achievement of feeding goals [18] because of the elevated intra-abdominal pressure and decreased gastrointestinal mobility caused by the heavy sedation induced by midazolam or propofol and the total paralysis caused by neuromuscular blocking agents [19]. One study discovered that EN was stopped more frequently for patients in the prone position than for those in the supine position [20]. Vomiting episodes were also more frequent for patients with ARDS receiving PP therapy [21]. Because prolonged PP therapy has become the standard of care for moderate to severe ARDS and CARDS, the aim of the current study investigated the association between EAR and ICU mortality in patients with high nutritional risk and ARDS receiving prolonged PP therapy.

## 2. Materials and Methods

### 2.1. Study Design and Patient Enrollment

**Figure 1:**
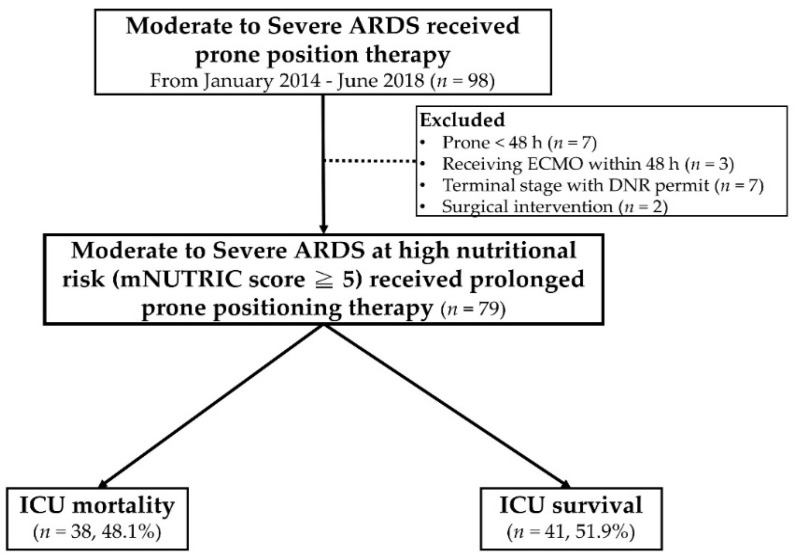
Study flowchart. ARDS: acute respiratory distress syndrome; ECMO: extracorporeal member oxygenation; DNR: do not resuscitate; ICU: intensive care unit.

This retrospective cohort study investigated the respiratory intensive care unit (RICU) of Taichung Veterans General Hospital (TCVGH), a tertiary referral center in Taiwan, from January 2014 to June 2018. We enrolled patients with high nutritional risk and a diagnosis of moderate to severe ARDS requiring mechanical ventilation and prolonged PP therapy for at least 48 h in the first week of ICU admission. High nutritional risk was defined as an mNUTRIC score of ≥5 in the first ICU stay or a feeding volume of <750 mL/day within 48 h of ICU admission, as in our previous publications [8,10]. Moderate to severe ARDS was defined as partial pressure of oxygen/fraction concentration of inspired oxygen ratio (PaO_2_/FiO_2_ ratio) of <150 mm Hg in accordance with the Berlin definition of ARDS [8,10,22,23]. The following patients were excluded: those requiring surgical intervention for acute abdominal infection or extracorporeal membrane oxygenation support within 48 h of admission because of failed PP therapy; those with comorbid poor cardiac function; those with active cancer in the terminal stage and a do not resuscitate order; and those who did not receive continuous PP therapy for more than 48 h (Figure 1). The demographic data, comorbidities, severity scores, daily feeding status, and clinical outcomes were extracted from electronic medical records. The study protocol was reviewed and approved by the Institutional Review Board of TCVGH (IRB number, CE20308B; date of approval, 16 September 2020). The study was conducted in accordance with the Declaration of Helsinki and relevant guidelines and regulations. The requirement for informed consent was waived because of the retrospective nature of the study, and the patients’ personal information was deidentified prior to analysis.

### 2.2. Protocol of Prone Positioning Therapy

The RICU is a 24-bed medical ICU that services adult patients with diagnoses of sepsis, acute respiratory failure, and ARDS requiring mechanical ventilation. For patients diagnosed as having moderate to severe ARDS, the standard of care in the RICU is to follow a lung protective strategy to maintain plateau pressure at less than 30 cm H_2_O by using low-tidal-volume ventilation (4–6 mL/kg) [14,24]. PP therapy is the first choice for rescue therapy in our RICU [25] when patients with moderate to severe ARDS experience refractory hypoxemia less than 24 h, a standard modified from that of landmark studies [14,15,26,27]. Since 2007, the protocol for PP therapy in our RICU was at least 48 h of continuous therapy [25,28]. Once the patients’ hypoxemia improves and their clinical condition stabilizes (i.e., when peripheral capillary oxygen saturation >90% and FiO_2_ <60% for >24 h after at least 48 h of PP therapy), patients are turned to the supine position. For prolonged PP therapy, patients require heavy sedation with medications such as midazolam or propofol and total paralysis through neuromuscular blocking agents to achieve a Richmond Agitation Sedation Scale score of less than −4. During PP therapy, patients are alternately turned right and left every 2 h to reduce the risk of pressure sore formation in the facial area, as described in our previous studies [25].

### 2.3. Protocol of Nutritional Risk Evaluation and Treatment

The evaluation of nutritional risk and suggestions for personalized nutritional prescriptions have been supported by a registered dietitian in our RICU since 2016. The mNUTRIC score and EAR were recorded, and nutritional prescriptions were suggested by the dietitian, as in our previous studies [8,9,10,11,29]. Early EN, the standard of care, was provided through a nasogastric tube on the first day of RICU admission for each patient, even for those requiring PP therapy [25]. The target energy requirement was 25–30 kcal/kg/day, and the target protein intake was 1.2 g/kg/day in accordance with the guidelines [3,4]. For patients who could not tolerate the standard feeding target, trophic feeding was provided to achieve a target of approximately 600 kcal/day, and 8–10 kcal/kg/day was also allowed during PP therapy [9].

### 2.4. Data Collection, Assessment, and Outcome Measures

Data on age, gender, body mass index (BMI), severity of illness score (Sequential Organ Failure Assessment [SOFA], Acute Physiology and Chronic Health Evaluation [APACHE] II, and mNUTRIC scores), major comorbidities, and PaO_2_/FiO_2_ ratio were extracted from the electronic medical records. The index date was the day of initiating PP therapy for ARDS. Daily EAR (%) was recorded on the index day and the seven days thereafter. The energy intake and energy intake achievement rate (%) of each day were calculated as follows: (actual energy intake/estimated energy requirement) × 100 [8,9,10,11]. The primary outcome was the correlation between energy intake achievement rate and ICU mortality. We identified an EAR of <65% in the first week of ICU admission as a poor prognostic factor for patients with high nutritional risk in our previous study [8,11], and this study was conducted to confirm the power of this predictor of ICU mortality.

### 2.5. Statistical Analysis

SPSS (version 22.0; International Business Machines Corp, Armonk, NY, USA) was used to perform the statistical analysis. The categorical variables are presented as frequencies and percentages. A chi-squared test was performed to determine significance. For nonparametric data distributions, a Mann–Whitney U test was performed to identify the differences between groups, and the results are presented as medians and interquartile ranges (IQRs). Cox regression analysis was performed to identify the factors associated with mortality. The strength of associations is presented with hazard ratios (HRs) and 95% confidence intervals (CIs). The survival curves were constructed through Kaplan–Meier analysis. A log-rank test was performed to identify significant differences in survival outcome between groups. All tests were two sided, with *p* < 0.05 considered significant.

## 3. Results

### 3.1. Patients’ Clinical and Demographic Characteristics

A total of 79 patients with moderate to severe ARDS receiving prolonged PP therapy (>48 h) were enrolled in this study (Figure 1). The median mNUTRIC score of this cohort was 7 (IQR: 5–8), indicating that the enrollees had high nutrition risk and required additional energy intake to reduce mortality [3,4]. The median APACHE II and SOFA scores were 31 (IQR: 27–33) and 10 (IQR: 8–14), respectively, and the median PaO_2_/FiO_2_ was 92.5 (IQR: 70.1–114.3), indicating high clinical severity, severe hypoxemia, and a higher probability of mortality. The overall mortality rate in the ICU was 48.1%. The average EAR (%) was higher during the post–PP therapy period than during PP therapy (64.5% and 42%). However, the median EAR (55.5%, IQR: 33.1–81.8%) was lower than 65% in the first seven days after the index date (Table 1).

### 3.2. Differences between Survival and Non-Survival Groups

**Figure 2:**
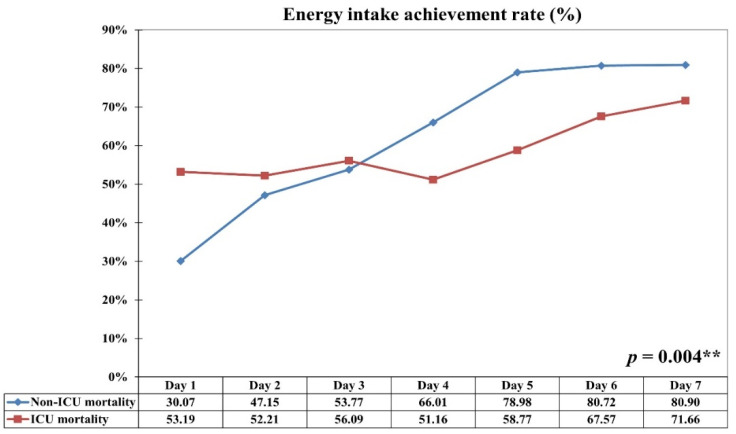
Comparison of energy achievement rate (EAR) during the first week of intensive care unit (ICU) admission between the survival and non-survival groups. ** *p* < 0.01.

Figure 2 presents a comparison of the EAR of the survival and non-survival groups in the first seven days of prolonged PP therapy. In the survival group, the EAR (%) significantly increased during the post–PP therapy period (days 4–7). However, only a minimal increase in EAR (%) was observed in the non-survival group. The survival group was significantly different from the non-survival group in terms of the distribution of the EAR (%) in the first seven days (*p* = 0.004; Figure 2).

The characteristics of the survival and non-survival groups were compared (Table 2). The non-survival group had a higher mNUTRIC score and lower EAR (%) in the post–PP therapy period (days 4–7). Significant differences were observed in age and number of patients who had renal replacement therapy (RRT) in the ICU and had comorbid active solid or hematologic malignancy between the survival and non-survival groups (all *p* < 0.05). For the survival group, the median EAR was 65% for days 4–7 (77.9%, IQR: 47.2–102.7%). In contrast, the median EAR (%) for the non-survival group was below 65% (51.1%, IQR: 26.6–87.4%), which was a significant difference (*p* = 0.025; Table 2). A significant difference was also observed on day 5 for the survival group compared with the non-survival group (73.8% and 47.0%, *p* = 0.033; Appendix A Table A1).

### 3.3. Factors Associated with ICU Mortality for Patients with ARDS Who Received PP Therapy

**Figure 3:**
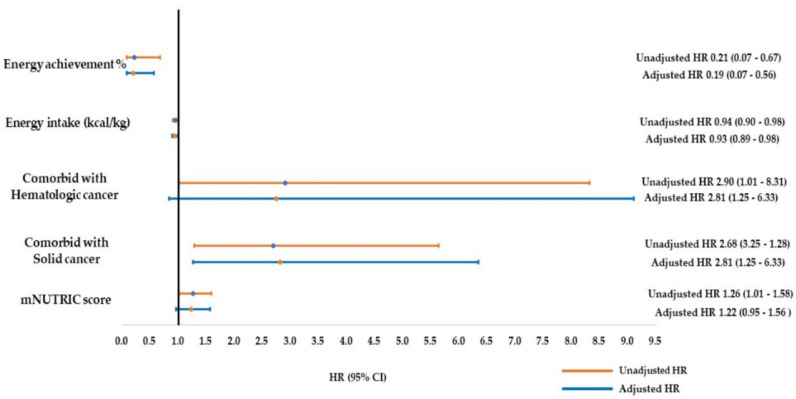
Hazard ratio (HR) of ICU mortality of critically ill patients with high nutritional risk and moderate to severe ARDS receiving prolong prone positioning (PP) therapy. mNUTRIC score: modified nutrition risk in the critically ill score.

**Figure 4:**
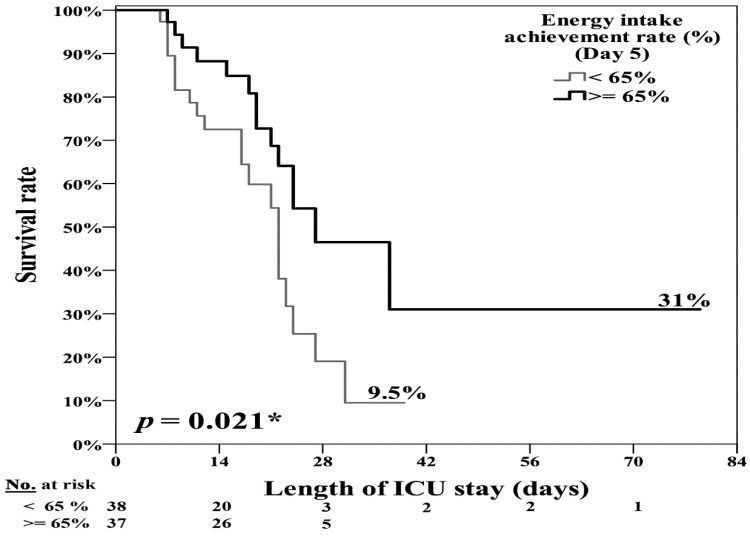
EAR >65% at the fifth ICU day was significantly associated with lower ICU mortality in patients with moderate to severe ARDS receiving prolonged PP therapy. * *p* < 0.05.

Table 3 and Figure 3 present the results of the Cox regression analysis of the factors associated with mortality in the ICU. Univariate analysis revealed five factors associated with mortality in the ICU: mNUTRIC score (HR: 1.26; 95% CI: 1.01–1.58; *p* = 0.038), comorbid active solid cancer (HR: 2.68; 95% CI: 1.28–5.62; *p* = 0.009) and hematologic malignancy (HR: 2.90; 95% CI: 1.01–8.31; *p* = 0.47), average energy intake (kcal/body weight; HR: 0.94; 95% CI: 0.90–0.98; *p* = 0.007), and EAR (%) (HR: 0.21; 95% CI: 0.07–0.64; *p* = 0.006) in the post–PP therapy period (days 4–7). Multivariate analysis revealed that a higher EAR (%) for post-admission days 4–7 (HR: 0.19; 95% CI: 0.07–0.56) was a strong predictive factor in the survival and non-survival groups (Table 3 and Figure 2). The EAR (%) on the fifth day after the initiation of PP therapy was significantly different between the survival and non-survival groups (Appendix A Table A1). Therefore, we used an EAR of >65% on the fifth post–PP therapy day as the cutoff value to create the Kaplan–Meier survival curves and perform the log-rank test on the survival and non-survival groups. An EAR of >65% was associated with lower 14-day, 28-day, and ICU mortality (*p* = 0.021) after adjustment for age, sex, BMI, and APACHE II and SOFA scores (Figure 4).

## 4. Discussion

This study yielded three major findings. First, ICU mortality was as high as 48.1% for patients with high nutritional risk and moderate to severe ARDS requiring prolonged PP therapy, even for those receiving EN within 24 h of admission. This high mortality may contribute to the severity of the disease and comorbidities, increase nutritional risk, and decrease EAR within seven days after initiating prolonged PP therapy. Second, although the average median EAR in the first seven days after PP therapy for the survival and non-survival groups was less than 65% (57.4% and 55.1%, respectively; *p* = 0.498), the EAR increased significantly in the survival group during PP therapy recovery (days 4–7). Third, an EAR of <65% on day 5 after prolonged PP therapy was an effective predictor of ICU mortality. To the best of our knowledge, this is the first study to evaluate the association between EAR and ICU mortality in patients with high nutritional risk and moderate to severe ARDS requiring prolonged PP therapy.

The prevalence of malnutrition and undernutrition is approximately 50–60% for critically ill patients admitted to the ICU. High nutritional risk is also correlated with morbidity and mortality in the ICU [30,31,32]. The standard to identify patients with malnourishment and high nutritional risk is uncertain in the current guidelines [1]. However, screening tools such as the Nutrition Risk Screening 2002 (NRS-2002), the nutrition risk in the critically ill (NUTRIC) and the mNUTRIC have been widely applied and recommended for use in the ICU [2,3,4]. The mNUTRIC score is a composite of five parameters: age, comorbidities, APACHE II score, SOFA score, and days in hospital before ICU admission [33]. Our previous study demonstrated that in critically ill patients, high nutritional risk (mNUTRIC score ≥ 5) was associated with higher ICU mortality [11]. Few studies have investigated the association between nutritional screening tools and clinical outcomes in critically ill patients with ARDS. One retrospective study conducted in South Korea proposed that the geriatric nutritional risk index (GNRI) is associated with 30-day mortality in elderly patients with ARDS [34]. However, another report noted the GNRI’s low specificity (57.1%) compared with the specificity of other nutritional indexes such as NRS 2002 and Onodera’s prognostic nutritional index for short-term outcomes in geriatric patients with respiratory failure [35]. In addition, the applicability of the GNRI may be limited because it is used to evaluate the geriatric population [36]. In our ICUs, the dietitian calculates the mNUTRIC score and feeding volume for all adult patients rather than only geriatric patients within 48 h to determine nutritional risk. Therefore, our study was the first to demonstrate that mNUTRIC score, rather than APACHE II or SOFA score, is significantly associated with ICU mortality for adult patients with ARDS requiring PP therapy. As our previous study also demonstrated [8,9,10,11], mNUTRIC score is a useful tool to evaluate nutritional risk in critically ill adult patients admitted to medical ICUs.

Several studies have proposed predictive factors associated with mortality in patients with ARDS requiring PP therapy [25,37,38]. However, few studies have addressed the effect of nutrition and the achievement of feeding goals on mortality. One retrospective study enrolled 43 patients who received PP therapy for ARDS and discovered three factors associated with mortality: APACHE II score, plateau pressure, and driving pressure in the lung mechanism [37]. Kao et al. retrospectively investigated factors associated with 60-day mortality in 65 patients with influenza-related ARDS who received PP therapy. The study identified higher pneumonia severity scores, increased driving pressure in the lung mechanism, and the comorbidity of requiring RRT [38]. Age, APACHE II score, malignant comorbidity, RRT requirement, and non-influenza-related ARDS were identified as predictive factors of ICU mortality in an investigation of 116 patients with severe ARDS requiring PP therapy [25]. However, the effects of nutritional support and the achievement of feeding goals in the first week of ICU admission on mortality in such patients were not considered. In the era of the COIVD-19 pandemic, PP therapy began to be widely recommended in treatment guidelines for patients with severe CARDS [16,17], and the crucial nature of nutrition support during PP therapy garnered attention [18,39,40]. This study identified two factors related to nutrition, namely mNUTRIC score (HR: 1.26; 95% CI: 1.01–1.58) and EAR (HR: 0.21; 95% CI, 0.07–0.64) on the fifth day after the initiation of PP therapy; this fills a gap in the research regarding the effect of nutritional support on ICU mortality for patients with ARDS requiring PP therapy.

This study demonstrated that even in patients with ARDS requiring a long period of PP therapy, an EAR >65% within the first week of ICU admission was associated with lower mortality risk in medical ICUs than that revealed in our previous studies [8,10,11]. The optimal EAR is 60–70% of the nutritional target in the first week in the ICU, as recommended by the 2016 ASPEN and SCCM guidelines and the 2019 ESPN guidelines [1,3,4]. PP therapy may be perceived as a barrier to providing early nutrition and achieving the energy target because of concerns regarding feasibility, safety, and tolerance. However, Reignier et al. revealed a significant improvement in feeding volume after a feeding protocol implementation in ARDS patients required PP therapy [41]. Because our RICU has evaluated nutritional risk and implemented the feeding protocol within 24 h of admission for all critically ill patients since 2016 [8,9,10,11], feeding targets are monitored and titrated to the maximum volume, even for patients requiring PP therapy. Therefore, our study also revealed that the difference in EAR on each day (Figure 2), rather than the average EAR in the first week of ICU admission, provides more information regarding mortality risk for patients receiving the feeding protocol. To the best of our knowledge, this study is the first to examine the EAR and its effect on ICU mortality in critically ill patients with moderate to severe ARDS requiring prolonged PP therapy.

This study had several limitations. First, the retrospective design limited the explanation of the results because of heterogeneity among the patients. Second, a single center, rather than multiple centers, was studied, which may have limited the generalizability of the results. Third, the enrollment of patients with moderate to severe ARDS may have confounded the ICU mortality risk. Because few studies have investigated the effect of nutritional support in patients with ARDS receiving PP therapy for at least 48 h, this study offers useful information for academic practice. Our RICU has practiced the standard protocol of lung protection, prolonged PP therapy [25], early EN within 24 h of admission, and feeding for all admitted critically ill patients with ARDS since 2007 [8,9,10,11]. Therefore, the limitations of the retrospective, single-center design should have been minimal. Although the severity of ARDS ranged from moderate to severe, the median PaO_2_/FiO_2_ ratio was less than 100 (median: 92.5; IQR: 70.1–114.3), and the difference in PaO_2_/FiO_2_ ratio was not significant in the univariate analysis of the Cox regression model. Therefore, the PaO_2_/FiO_2_ ratio was unlikely to be a confounding factor in the prediction of ICU mortality. Finally, our results may not be generalizable to critically ill patients in neurosurgical, surgical, cardiac, and pediatric ICUs because only adult patients admitted to a medical ICU were enrolled.

## 5. Conclusions

ICU mortality is high for adult patients with ARDS requiring PP therapy. The only score significantly associated with ICU mortality was mNUTRIC. An EAR of <50% was observed in both the survival and non-survival groups during PP therapy. However, only the survival group exhibited a significant increase in EAR during recovery from prolonged PP in the supine position (days 4–7 after initiation of PP therapy). The key factor in determining ICU mortality in this population was an EAR of <65% by day 5 after the initiation of prolonged PP therapy. For patients with high nutrition risk (mNUTRIC score ≥ 5) and moderate to severe ARDS requiring prolonged PP therapy, we suggest early EN and increasing the feeding volume to the goal of >65% during the first week of ICU admission. For patients with an EAR of <65% during the first week, nutrition support therapy with postpyloric tube placement or partial parenteral nutrition is required.

## Figures and Tables

**Table 1:** Demographic characteristics, severity scores, comorbidities, and clinical outcomes of patients with moderate to severe ARDS receiving prolonged PP therapy in the ICU.

Characteristics	Median (IQR) or *n* (%) (*n* = 79)
Demographic data	
Age (y/o) (*n*, %)	61.5 (51.1–74)
Gender-Male (*n*, %)	48 (60.8%)
mNUTRIC score	7.0 (5–8)
APACHE II score	31.0 (27–33)
SOFA	10.0 (8–14)
Renal replacement therapy (*n*, %)	35 (44.30%)
Comorbidity	
CAD (*n*, %)	6 (7.59%)
COPD (*n*, %)	10 (12.66%)
Solid cancer (*n*, %)	11 (13.92%)
Hematologic malignancies (*n*, %)	5 (6.33%)
DM (*n*, %)	24 (30.38%)
CKD (*n*, %)	27 (34.18%)
Autoimmune disease (*n*, %)	12 (15.19%)
PaO_2_/FiO_2_ (PF ratio)	92.5 (70.1–114.3)
Actual energy intake (kcal/BW)	
During prolonged PP (d 1–d 3)	7.9 (4.6–13)
Post prolonged PP (d 4–d 7)	12.0 (7.3–18.8)
Average in the first 7 d	10.8 (6.6–15.2)
Energy achievement rate (%)	
During prolonged PP (d 1–d 3)	42.0 (23.8–64.9)
Post prolonged PP (d 4–d 7)	64.5 (36.4–91.8)
Average in the first 7 d	55.5 (33.1–81.8)
ICU mortality (*n*, %)	38 (48.10%)

Continuous data are expressed as medians and interquartile ranges (IQRs). Categorical data are expressed as numbers and percentages. APACH II: acute physiology and chronic health evaluation II; SOFA: Sequential Organ Failure Assessment; CAD: coronary artery disease; COPD: chronic obstructive pulmonary disease; DM: diabetes mellitus; CKD: chronic kidney disease. mNUTRIC: modified nutrition risk in the critically ill; PP: prone positioning; PF ratio: partial pressure of oxygen/fraction concentration of inspired oxygen ratio.

**Table 2:** Demographic characteristics, severity index, comorbidities, and EAR in survival and non-survival groups.

Characteristics	Survival (*n* = 41)	Non-Survival (*n* = 38)	*p* Value
Demographic data			
Age (y/o) (*n*, %)	56.8 (46–68.3)	63.8 (56.9–76.5)	0.036 *
Gender-Male (*n*, %)	27 (65.9%)	21 (55.3%)	0.464
mNUTRIC score	6.0 (4–7)	7.0 (6–8)	0.002 **
APACHE II score	31.0 (26.5–32.5)	31.0 (26.8–34.3)	0.470
SOFA	10.0 (8–14.5)	10.5 (8–14.3)	0.996
Renal replacement therapy (*n*, %)	13 (31.7%)	22 (57.9%)	0.034 *
Comorbidity			
CAD (*n*, %) ^f^	3 (7.3%)	3 (7.9%)	1.000
COPD (*n*, %) ^f^	6 (14.6%)	4 (10.5%)	0.739
Solid cancer (*n*, %)	1 (2.4%)	10 (26.3%)	0.006 **
Hematologic malignancies (*n*, %)	1 (2.4%)	4 (10.5%)	0.190
DM (*n*, %)	16 (39.0%)	8 (21.1%)	0.136
CKD (*n*, %)	12 (29.3%)	15 (39.5%)	0.473
Autoimmune disease (*n*, %)	5 (12.2%)	7 (18.4%)	0.648
PaO_2_/FiO_2_ (PF ratio)	96.5 (73.2–125.2)	88.7 (65.5–104.3)	0.133
Actual energy intake (kcal/BW)			
During prolonged PP (d 1–d 3)	6.5 (4–11.6)	9.2 (5.1–14.8)	0.133
Post prolonged PP (d 4–d 7)	12.8 (9–21.2)	10.2 (5.3–16.9)	0.049 *
Average in the first 7 d	10.5 (7.3–16.4)	10.9 (5.6–15.5)	0.638
Energy achievement rate (%)			
During prolonged PP (d 1–d 3)	39.3% (19.8–59.4%)	46.1% (29.2–76.0%)	0.192
Post prolonged PP (d 4–d 7)	77.9% (47.2–102.7%)	51.1% (26.6–87.4%)	0.025 *
Average in the first 7 d	57.4% (37.3–82.1%)	55.1% (28.2–82.1%)	0.498

Mann–Whitney U test. Chi-square test. ^f^ Fisher’s exact test. * *p* < 0.05, ** *p* < 0.01. Continuous data are expressed as medians and IQRs. Categorical data are expressed as numbers and percentages. APACH II: acute physiology and chronic health evaluation II; SOFA: Sequential Organ Failure Assessment; CAD: coronary artery disease; COPD: chronic obstructive pulmonary disease; DM: diabetes mellitus; CKD: chronic kidney disease. mNUTRIC: modified nutrition risk in the critically ill; PP: prone positioning; PF ratio: partial pressure of oxygen/fraction concentration of inspired oxygen ratio.

**Table 3:** Univariate and multivariate analyses of factors associated with ICU mortality.

Characteristics	Univariate Analysis HR (95% CI) *p* Value		Multivariate Analysis HR (95% CI) *p* Value	
Demographic data				
Age	1.02 (1.00–1.04)	0.062		
Sex (Female/Male)	0.76 (0.40–1.44)	0.401		
BMI (kg/m^2^)	1.01 (0.94–1.08)	0.848		
mNUTRIC score	1.26 (1.01–1.58)	0.038 *	1.22 (0.95 0.56)	0.116
APACHE II score	1.04 (0.98–1.09)	0.182		
SOFA	1.03 (0.95–1.12)	0.510		
Renal replacement therapy (*n*, %)	1.31 (0.68–2.50)	0.422		
Comorbidity				
CAD (*n*, %) ^f^	1.32 (0.40–4.32)	0.648		
COPD (*n*, %) ^f^	0.65 (0.23–1.86)	0.426		
Solid cancer (*n*, %)	2.68 (1.28–5.62)	0.009 **	2.81 (1.25–6.33)	0.013 *
Hematologic malignancies (*n*, %)	2.90 (1.01–8.31)	0.047 *	2.74 (0.83–9.10)	0.099
DM (*n*, %)	1.03 (0.46–2.28)	0.945		
CKD (*n*, %)	1.15 (0.60–2.22)	0.668		
Autoimmune disease (*n*, %)	1.09 (0.48–2.48)	0.839		
PaO_2_/FiO_2_ (PF ratio)	0.99 (0.98–1.00)	0.084		
Actual energy intake (kcal/BW)				
During prolonged PP (d 1–d 3)	1.00 (0.96–1.04)	0.994		
Post prolonged PP (d 4–d 7)	0.94 (0.90–0.98)	0.007 **	0.93 (0.89–0.98)	0.006 **
Average in the first 7 d	0.97 (0.92–1.01)	0.144		
Energy achievement rate (%)				
During prolonged PP (d 1–d 3)	1.00 (0.38–2.59)	0.994		
Post prolonged PP (d 4–d 7)	0.21 (0.07–0.64)	0.006 **	0.19 (0.07–0.56)	0.002 **
Average in the first 7 d	0.42 (0.14–1.27)	0.124		

Cox regression. * *p* < 0.05, ** *p* < 0.01. APACH II: acute physiology and chronic health evaluation II; SOFA: Sequential Organ Failure Assessment; CAD: coronary artery disease; COPD: chronic obstructive pulmonary disease; DM: diabetes mellitus; CKD: chronic kidney disease. mNUTRIC: modified nutrition risk in the critically ill; PP: prone positioning; PF ratio: partial pressure of oxygen/fraction concentration of inspired oxygen ratio. ^f^ Fisher’s exact test.

## Data Availability

The data presented in this study are available on request from the corresponding author. The data are not publicly available due to the regulation of Institutional Review Board of Taichung Veterans General Hospital in Taiwan.

## Appendix A

**Table A1:** Comparison of EAR between survival and non-survival groups during the first week of ICU admission.

Characteristics	Survival (*n* = 41)		Mortality (*n* = 38)		*p* Value
	%	IQR	%	IQR	
Energy achievement rate (%)					
day 1	18.3%	(7.3–46.8%)	50.1%	(17.0–79.8%)	0.010 *
day 2	46.8%	(19.1–71.3%)	47.4%	(22.4–77.1%)	0.603
day 3	49.9%	(23.4–74.5%)	46.6%	(28.4–76.0%)	0.791
day 4	69.9%	(40.7–86.1%)	39.3%	(25.5–73.6%)	0.052
day 5	73.8%	(44.0–112.4%)	47.0%	(30.7–83.0%)	0.033 *
day 6	73.1%	(45.6–115.5%)	65.2%	(24.6–98.5%)	0.185
day 7	72.3%	(52.1–105.3%)	76.0%	(24.0–101.2%)	0.388

Mann–Whitney *U* test. Chi-square test. * *p* < 0.05.
